# Apolipoprotein A-II induces acute-phase response associated AA amyloidosis in mice through conformational changes of plasma lipoprotein structure

**DOI:** 10.1038/s41598-018-23755-y

**Published:** 2018-04-04

**Authors:** Mu Yang, Yingye Liu, Jian Dai, Lin Li, Xin Ding, Zhe Xu, Masayuki Mori, Hiroki Miyahara, Jinko Sawashita, Keiichi Higuchi

**Affiliations:** 1 Department of Aging Biology, Institute of Pathogenesis and Disease Prevention, Shinshu University Graduate School of Medicine, Matsumoto, 290-8621 Japan; 2 Department of Molecular and Cellular Biology, Baylor College of Medicine, Houston, TX 77030 USA; 3 Institute of Pediatric Research, Children’s Hospital of Hebei Province, Shijiazhuang, 050031 China; 4 Department of Advanced Medicine for Health Promotion, Institute for Biomedical Sciences, Interdisciplinary Cluster for Cutting Edge Research, Shinshu University, Matsumoto, 290-8621 Japan; 5 Department of Biological Science for Intractable Neurological Disease, Institute for Biomedical Sciences, Interdisciplinary Cluster for Cutting Edge Research, Shinshu University, Matsumoto, 390-8621 Japan

## Abstract

During acute-phase response (APR), there is a dramatic increase in serum amyloid A (SAA) in plasma high density lipoproteins (HDL). Elevated SAA leads to reactive AA amyloidosis in animals and humans. Herein, we employed apolipoprotein A-II (ApoA-II) deficient *(Apoa2*^*−/−*^) and transgenic (*Apoa2*Tg) mice to investigate the potential roles of ApoA-II in lipoprotein particle formation and progression of AA amyloidosis during APR. AA amyloid deposition was suppressed in *Apoa2*^*−/−*^ mice compared with wild type (WT) mice. During APR, *Apoa2*^*−/−*^ mice exhibited significant suppression of serum SAA levels and hepatic *Saa1* and *Saa2* mRNA levels. Pathological investigation showed *Apoa2*^*−/−*^ mice had less tissue damage and less inflammatory cell infiltration during APR. Total lipoproteins were markedly decreased in *Apoa2*^*−/−*^ mice, while the ratio of HDL to low density lipoprotein (LDL) was also decreased. Both WT and *Apoa2*^*−/−*^ mice showed increases in LDL and very large HDL during APR. SAA was distributed more widely in lipoprotein particles ranging from chylomicrons to very small HDL in *Apoa2*^*−/−*^ mice. Our observations uncovered the critical roles of ApoA-II in inflammation, serum lipoprotein stability and AA amyloidosis morbidity, and prompt consideration of therapies for AA and other amyloidoses, whose precursor proteins are associated with circulating HDL particles.

## Introduction

Amyloidosis is a group of diseases characterized by extracellular or intracellular deposition of insoluble amyloid fibrils, which are aggregates formed from normally soluble proteins via conformational changes caused by various mechanisms^1,2^. Several serious human diseases such as Alzheimer’s disease, type II diabetes, prion disease and familial amyloid polyneuropathy (FAP) are associated with amyloid fibril deposition^3^. Reactive amyloid A (AA) amyloidosis is a systemic type of amyloidosis and occurs in domestic, laboratory and wild animals, as well as in humans^4–6^. AA amyloidosis is a major complication of chronic inflammation in patients with rheumatoid arthritis and serious infection diseases. As an acute phase plasma protein predominantly synthesized in the liver^7,8^, serum amyloid A (SAA) is deposited extracellularly as amyloid fibrils, which leads to tissue structure damage and dysfunction of various organs, including the liver, spleen, kidney and heart, among others^9,10^.

SAA was first identified as a serum protein that cross-reacts with antibodies against AA protein^11–13^. During the acute phase reaction (APR) of inflammation, the concentration of plasma SAA, as a high density lipoprotein (HDL) associated apolipoprotein, may increase up to ~1000-fold. SAA is an evolutionarily conserved protein, but its function has not been completely elucidated. As a biomarker for inflammation, its role in cancer, cardiovascular disease, and inflammatory processes remains controversial^14,15^. However, its adverse role has been established in the pathogenesis of AA amyloidosis. Sustained high levels of SAA result in tissue deposition of the N-terminal fragments of SAA as amyloid fibrils. In mice, AA amyloid deposition can be experimentally induced by multiple injections of silver nitrate (AgNO_3_), casein or lipopolysaccharide (LPS), resulting in remarkable elevation and maintenance of high levels of plasma SAA^16^. Amyloid enhancing factor (AEF) and AA amyloid fibrils have been used to induce and/or accelerate AA amyloidosis in mice and other animals, such as hens and rabbits^17–19^.

In addition to the stimulation of reverse cholesterol transport from extra-hepatic tissue to the liver, HDL is known for its preventive roles in cardiovascular disease through anti-oxidant and anti-inflammatory effects^20,21^. Apolipoprotein A-II (ApoA-II) is the second most abundant protein component of HDL; however, its roles in HDL function and metabolism remain unclear^22^. ApoA-II is reported to be more hydrophobic than apolipoprotein A-I (ApoA-I), and is closely associated with modulation of HDL metabolism and alteration of HDL conformation by interacting with ApoA-I and other apolipoproteins^23–25^. In mice, ApoA-II is a serum precursor of amyloid fibrils (AApoAII) in age-associated systemic amyloidosis (AApoAII amyloidosis)^26,27^. Our previous study found that mouse SAA, ApoA-I and ApoA-II interact with each other during AA and AApoAII amyloidosis^28,29^. It has been reported that during APR, elevated SAA binds to HDL and decreases levels of ApoA-I and ApoA-II, leading to alteration of HDL particle size^11,30,31^.

To investigate the potential role of ApoA-II in lipoprotein particle distribution and progression of AA amyloidosis, we induced AA amyloidosis by co-injection of AA amyloid fibrils (AEF) and AgNO_3_ (inflammation inducer) in wild type (WT), ApoA-II deficient (*Apoa2*^*−/−*^), ApoA-II overexpressing (*Apoa2*^*c*^Tg) and ApoA-I deficient (*Apoa1*^*−/−*^) mice. We found that elevation in serum SAA and AA amyloid deposition was significantly suppressed in *Apoa2*^*−/−*^ mice. Moreover, ApoA-II deficiency resulted in dramatic alteration of lipoprotein particles and redistribution of apolipoprotein in lipoproteins. These results suggest an important role of ApoA-II in inflammation, lipoprotein metabolism and AA amyloidosis.

## Results

### AA amyloid deposition was suppressed in *Apoa2*^*−/−*^ mice

**Figure 1:**
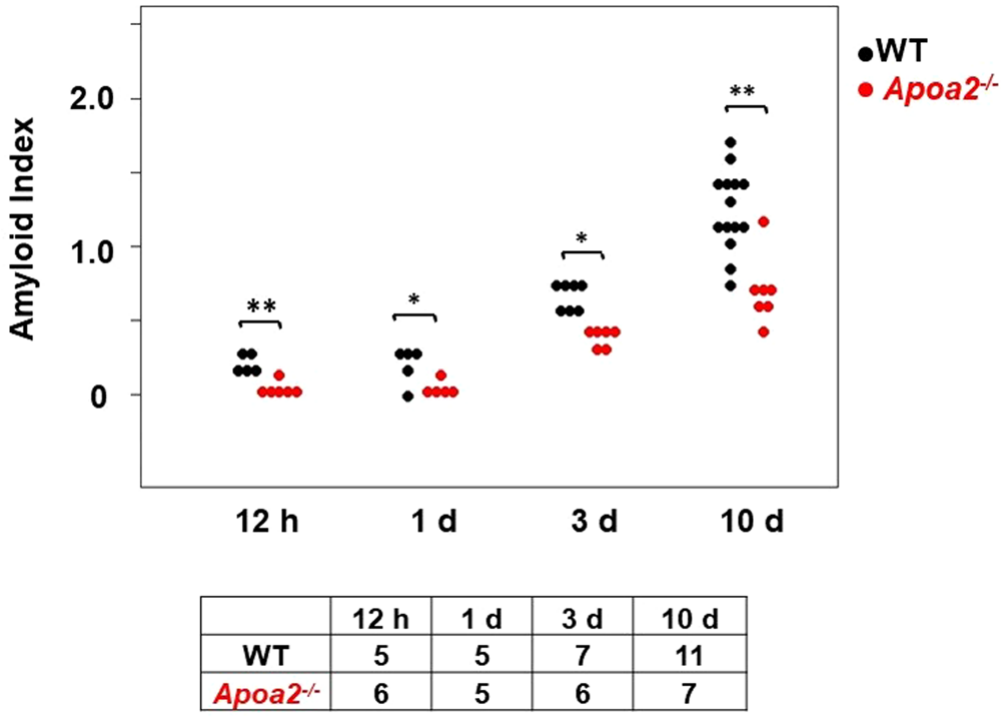
The degree of amyloid deposition in whole body (AI) was determined in WT and *Apoa2*^*−/−*^ mice at 12 h, 1 d, 3 d and 10 d after co-injection of AgNO_3_ and AA fibrils. The AI of each mouse is presented (●; WT and ; *Apoa2*^*−/−*^). AI was significantly lower in *Apoa2*^*−/−*^ mice than WT mice at 12 h to 10 d. *, ** significantly different between WT and *Apoa2*^*−/−*^ mice, P < 0.05, P < 0.01 (Mann-Whitney U-test). The table below the figure represents the number of mice used.

**Figure 2:**
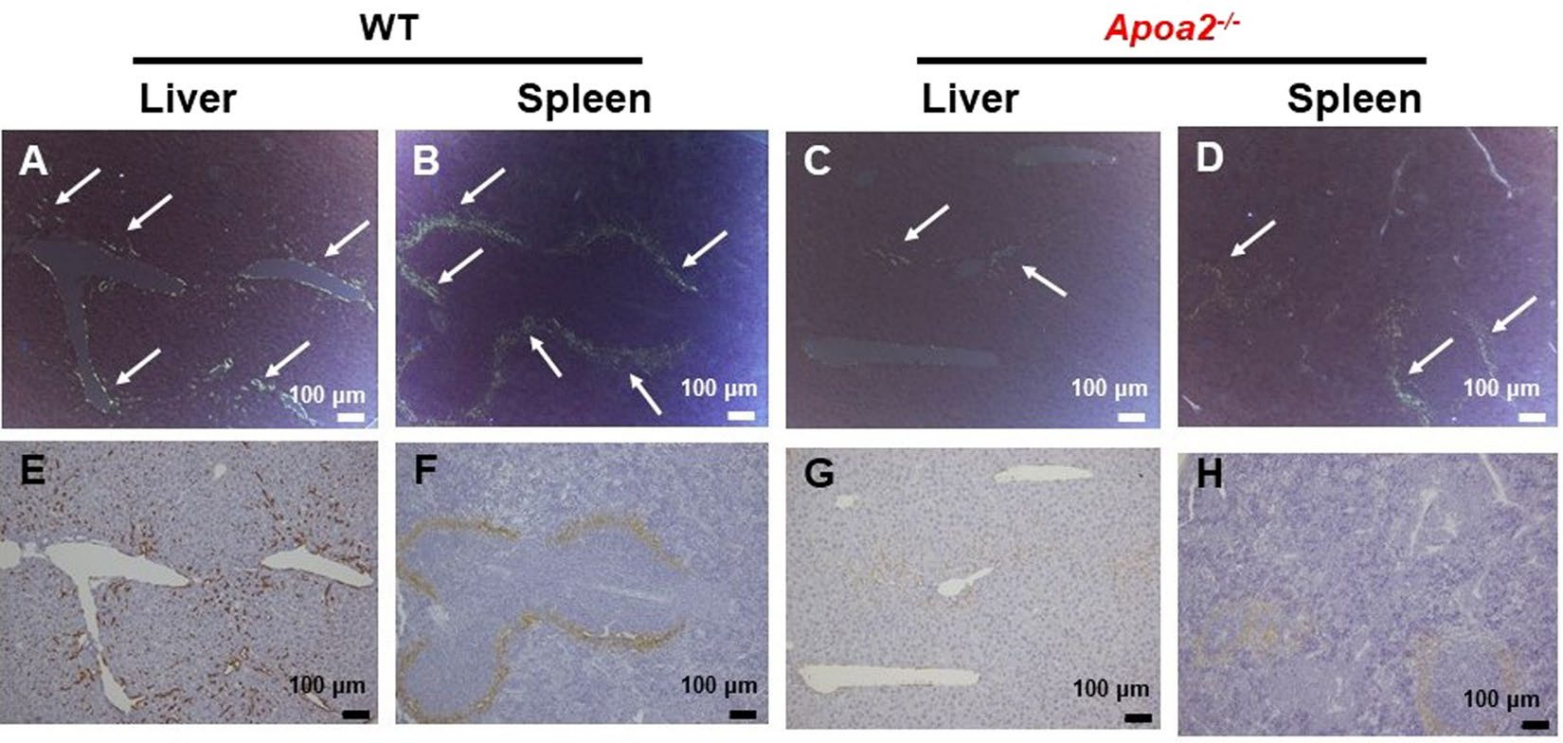
Amyloid deposition observed in the liver and spleen 10 d after co-injection with AgNO_3_ and AA fibrils. Amyloid deposition was detected by green birefringence in Congo red stained tissue (**A**–**D**) or brown positive area with antiserum against AA in immunohistochemically stained tissue (**E**–**H**) of WT (**A**,**B**,**E** and **F**) and *Apoa2*^*−/−*^ (**C**,**D**,**G** and **H**) mice.

After co-injection of AgNO_3_ and AA fibrils, tissue sections from various organs were stained with Congo red, and amyloid deposition (amyloid score: AS and amyloid index: AI) was subsequently determined by green birefringence under polarizing microscopy. The liver of WT and *Apoa2*^*−/−*^ mice and the spleen of WT mice showed AA amyloid deposition 12 h and 1 d after injection (Fig. 1 and Supplementary Fig. 1). After 3 to 10 d of injection, AA amyloid deposition had expanded from the liver and spleen to the stomach, intestine, lung and kidney (Fig. 2). ASs in these organs were significantly less in *Apoa2*^*−/−*^ mice than in WT mice (Figs 1 and 2). The degree of amyloid deposition in the whole body (AI) was significantly reduced in *Apoa2*^*−/−*^ mice at 12 h, 1 d, 3 d and 10 d after treatment (Fig. 1).

### Elevation of serum and hepatic SAA mRNA expression was suppressed in *Apoa2*^*−/−*^ mice

**Figure 3:**
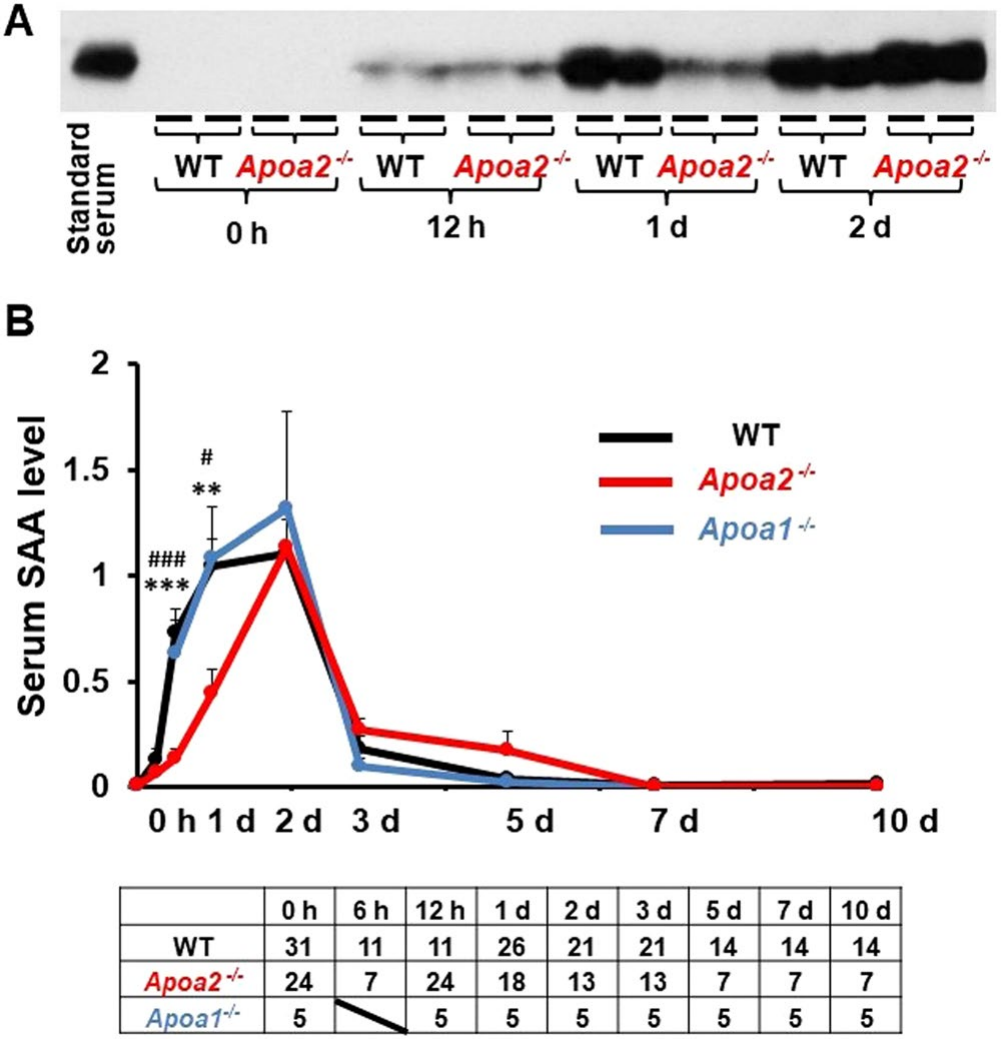
Serum SAA levels were elevated during induction of AA amyloidosis. WT, *Apoa2*^*−/−*^ and *Apoa1*^*−/−*^ mice were co-injected with AgNO_3_ and AA fibrils and serum were obtained at 0 h, 6 h, 12 h, 1 d, 3 d, 7 d and 10 d after injection from WT mice, *Apoa2*^*−/−*^ and *Apoa1*^*−/−*^ mice. Serum SAA levels were detected in WT and *Apoa2*^*−/−*^ mice with specific antisera following SDS-PAGE and Western blot (**A**). The serum concentrations of SAA were quantitated using a densitometric image analyzer with NIH Image, and serum SAA levels were represented as ratios to pooled standard serum isolated from C57BL/6 J mice 1 d after co-injection with AgNO_3_ and AA fibrils (**B**). Each symbol represents mean ± SEM. **P < 0.01, ***P < 0.001, WT vs *Apoa2*^*−/−*^, ^#^P < 0.05, ^###^P < 0.001, *Apoa1*^*−/−*^ vs *Apoa2*^*−/−*^ (Turkey–Kramer method of multiple comparisons at corresponding time). The table below the figure represents the number of mice used.

In WT and *Apoa2*^*−/−*^ mice, serum SAA levels were undetectable at 0 h, but increased dramatically and reached maximum levels 2 d after co-injection with AgNO_3_ and AA fibrils, and then deceased rapidly until being undetectable at 10 d. In contrast, upregulation of serum SAA was significantly suppressed at 12 h and 1 d after inflammatory stimulus in *Apoa2*^*−/−*^ mice compared with WT mice (Fig. 3). On the other hand, *Apoa1*^*−/−*^ mice showed no difference compared with WT mice (Fig. 3).

**Figure 4:**
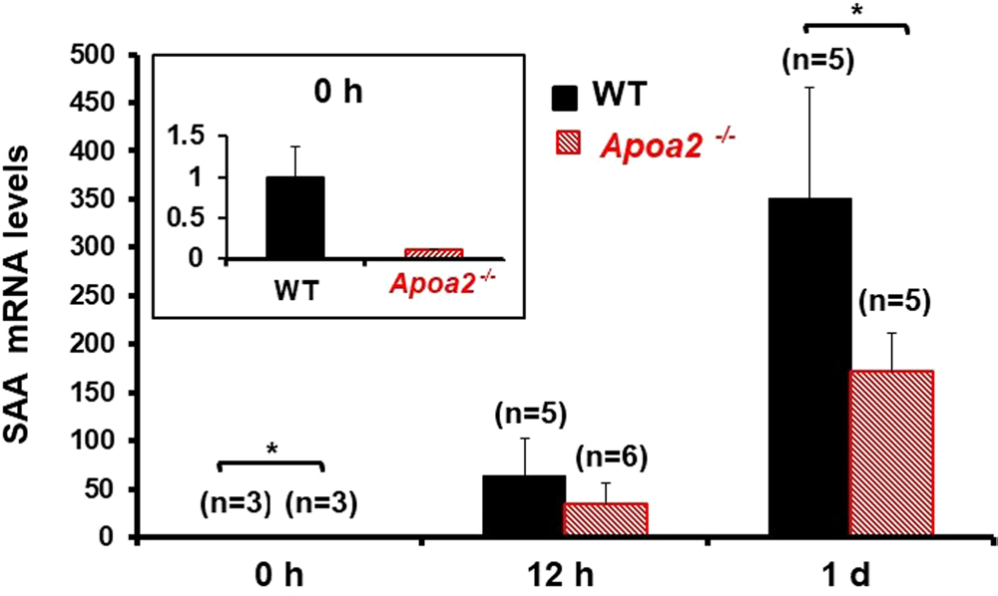
Suppressed hepatic *Saa1/Saa2* mRNA expression levels in *Apoa2*^*−/−*^ mice during the acute inflammation stage. mRNA expression levels of *Saa1/Saa2* in the liver of WT and *Apoa2*^*−/−*^ mice during the pre (0 h) and acute inflammation (12 h and 1 d) stages were determined by real time-PCR analysis. Expression levels are represented as ratios to a WT mouse at 0 h. Hepatic mRNA expression levels of SAA were suppressed in *Apoa2*^*−/−*^ mice compared with WT mice in the pre-inflammation stage. During acute inflammation, expression rates increased dramatically, by as much as ~350 times in WT mice, but were suppressed (~170 times) in *Apoa2*^*−/−*^ mice at 1 d after injection. Each column and bar represents mean ± SEM. *P < 0.05, WT vs *Apoa2*^*−/−*^ (unpaired Student’s t test with Bonferroni Correction for multiple comparisons).

To elucidate the mechanism leading to low serum SAA levels in *Apoa2*^*−/−*^ mice, real-time PCR was performed to assess hepatic *Saa1/Saa2* mRNA in WT and *Apoa2*^*−/−*^ mice (Fig. 4). Prior to experimental manipulation, *Apoa2*^*−/−*^ mice expressed a lower *Saa1/Saa2* mRNA level compared with WT (P < 0.05; Fig. 4). Furthermore, *Apoa2*^*−/−*^ mice also showed significantly lower SAA levels under inflammatory stimuli at 1 d (P < 0.05).

### Pathological damage in lungs was suppressed in *Apoa2*^*−/−*^ mice

**Figure 5:**
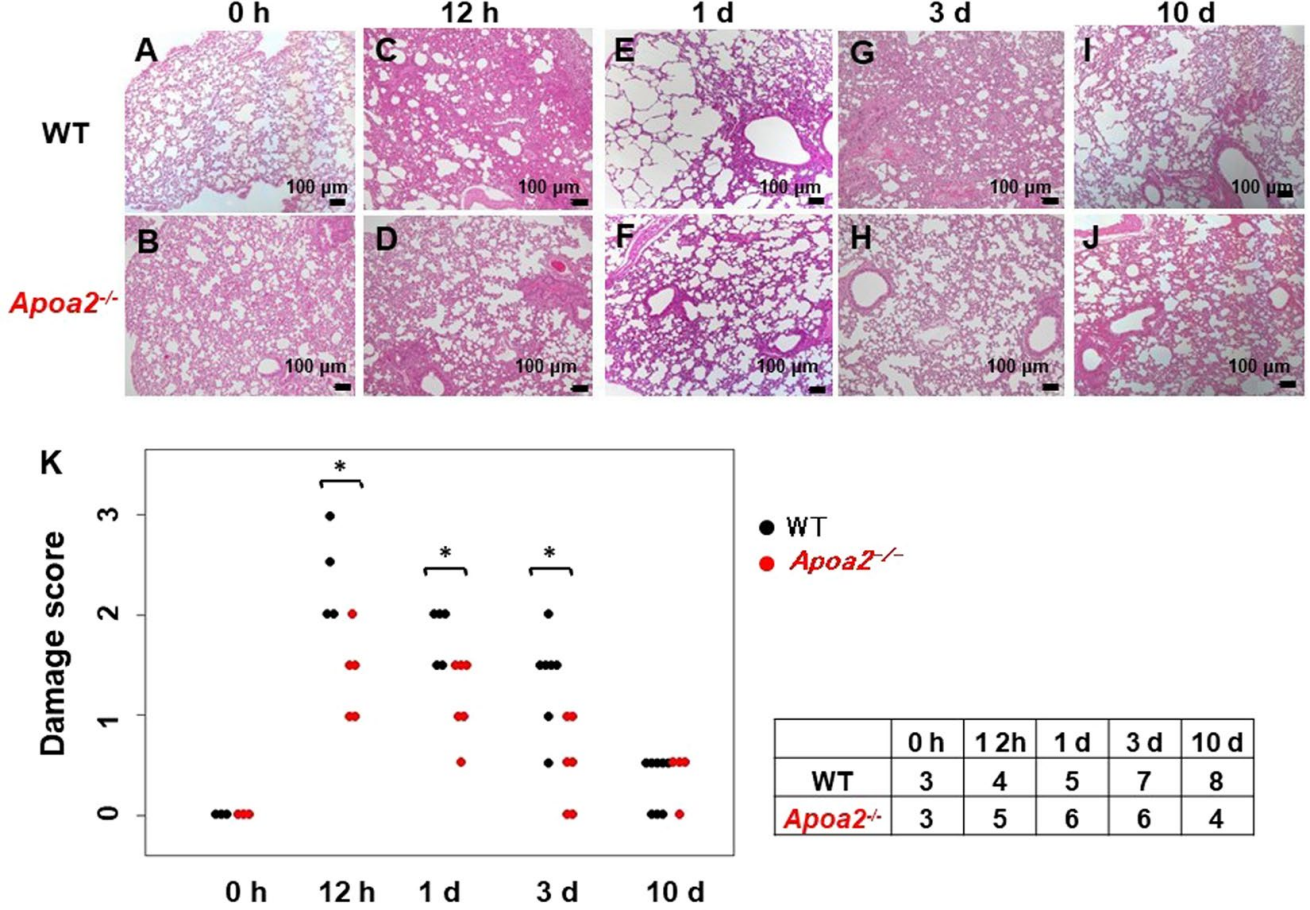
Pathological damage was examined in the lungs of WT (**A**,**C**,**E**,**G** and **I**) and *Apoa2*^*−/−*^ (**B**,**D**,**F**,**H** and **J**) mice during inflammation caused by co-injection with AgNO_3_ and AA fibrils. The level of pathological damage in the lungs of *Apoa2*^*−/−*^ mice was less severe than that of WT mice (HE × 100). Each scale bar indicates 100 μm. The damage score of each mouse is presented (**K**). There are significant differences in damage score between WT and *Apoa2*^*−/−*^ mice. *P < 0.05, significantly different between WT and *Apoa2*^*−/−*^ mice (Mann-Whitney U-test). The table on the right side of the figure represents the number of mice used.

Lungs of *Apoa2*^*−/−*^ and WT mice at 12 h to 10 d after treatment with AgNO_3_ and AA fibrils were evaluated microscopically. *Apoa2*^*−/−*^ mice experienced less tissue damage and less inflammatory cell infiltrates compared to WT mice at 12 h during APR (Fig. 5C and D). At 1 d during APR, *Apoa2*^*−/−*^ mice had less emphysematous changes than WT mice (Fig. 5E and F). At 3 d during APR, the lungs of WT still exhibited infiltration of inflammatory cells, while *Apoa2*^*−/−*^ mice demonstrated recovery from damage (Fig. 5G and H). At 10 d after APR, both WT and *Apoa2*^*−/−*^ mice showed recovery from inflammatory damage (Fig. 5I and J). Significant differences in damage scores were noted between WT and *Apoa2*^*−/−*^ mice at 12 h to 3 d (Fig. 5K).

### AA amyloid deposition and elevated serum SAA levels were accelerated in *Apoa2*Tg mice

**Figure 6:**
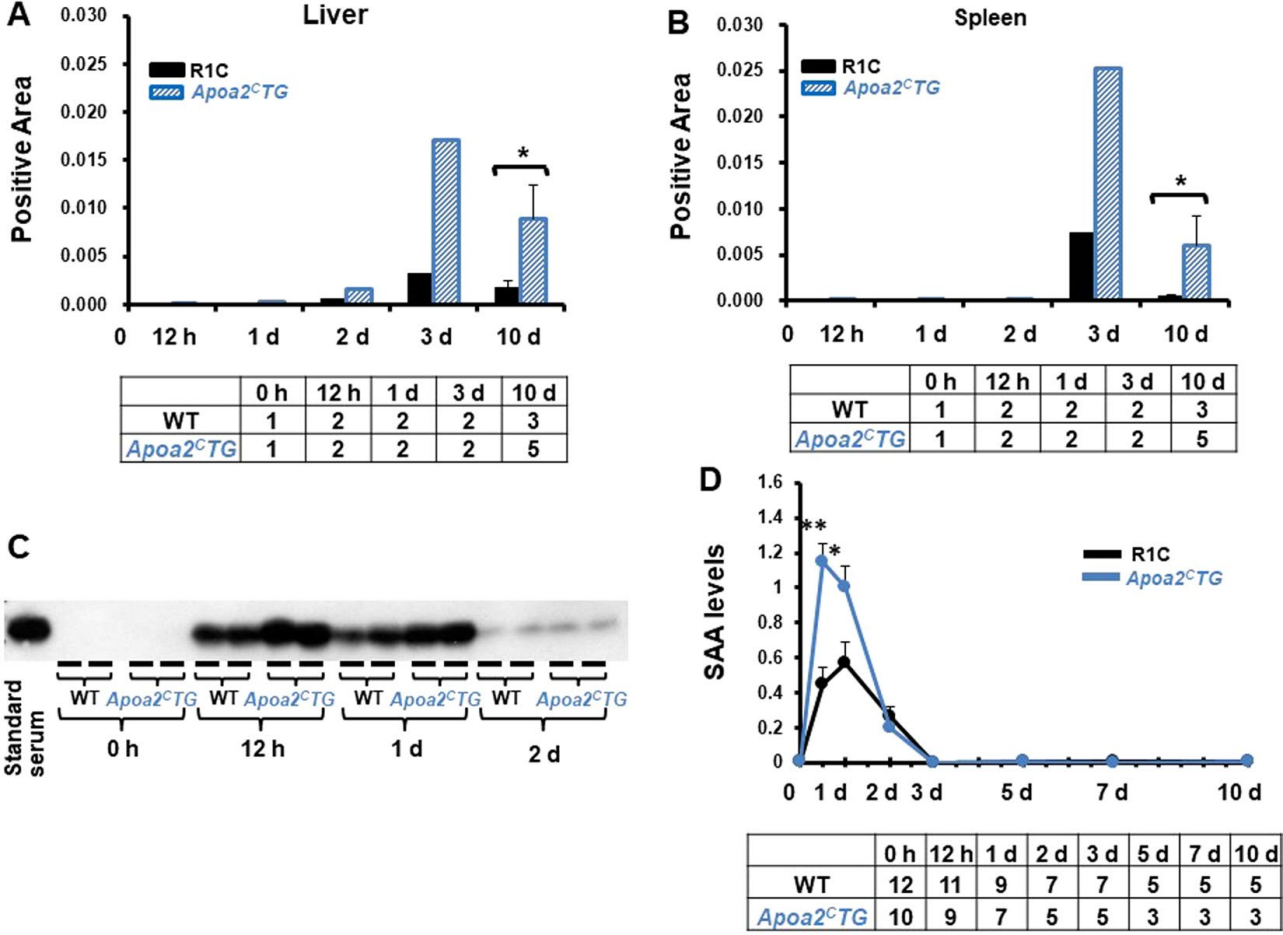
AA amyloid deposition and serum SAA levels were accelerated in *Apoa2*Tg mice. The positive areas of amyloid deposits in the liver and spleen were determined as IHC positive area with antiserum against AA at 3 and 10 d after co-injection with AgNO_3_ and AA fibrils (**A** and **B**). Three areas in each of the liver and spleen sections were randomly captured under x200 magnification, and the positive areas (ratio to whole liver and spleen) were calculated with the ImageJ software. Each column represents the mean at 12 h, 1 d, 3 d, and each column and bar represents the mean ± SEM. The tables below the figures represent the number of mice used. *P < 0.05, WT vs. *Apoa2*Tg (unpaired Student’s t-test). WT and *Apoa2*Tg mice were co-injected with AgNO_3_ and AA fibrils. Serum was obtained at 0 h, 12 h, 1 d, 2 d, 3 d, 5 d, 7 d and 10 d after injection, and serum SAA levels were determined by Western blot analysis (C and D). Serum SAA levels are represented as a ratio to pooled standard serum isolated from C57BL/6 J mice. Each symbol represents mean ± SEM. *P < 0.05, **P < 0.01, WT vs *Apoa2*Tg (unpaired Student’s t test). The table below the figure represents the number of mice used.

We compared AA amyloid deposition and serum SAA levels in WT (R1.P1-*Apoa2*^*c*^) mice with those in *Apoa2*Tg mice. The serum concentration of ApoA-II in *ApoA2*Tg was 1.26 times that in WT mice^32^. In *Apoa2*Tg mice, AA immunohistochemistry (IHC) positive area was more abundant in the liver and spleen at 3 d and 10 d after co-injection compared with WT mice. Significant differences at 10 d were noted in the liver and spleen (Fig. 6A and B). The amyloid deposition stained positively in IHC with anti-AA antiserum, but negatively with anti-AApoAII antiserum (data not shown). *Apoa2*Tg mice also showed significantly higher serum SAA levels at 12 h and 1 d after co-injection compared with WT mice (Fig. 6C and D).

These results suggest that increased levels of serum ApoA-II may accelerate the APR associated elevation of serum SAA levels and accelerate AA deposition.

### Lipoprotein particles exhibited smaller sizes in *Apoa2*^*−/−*^ mice

**Figure 7:**
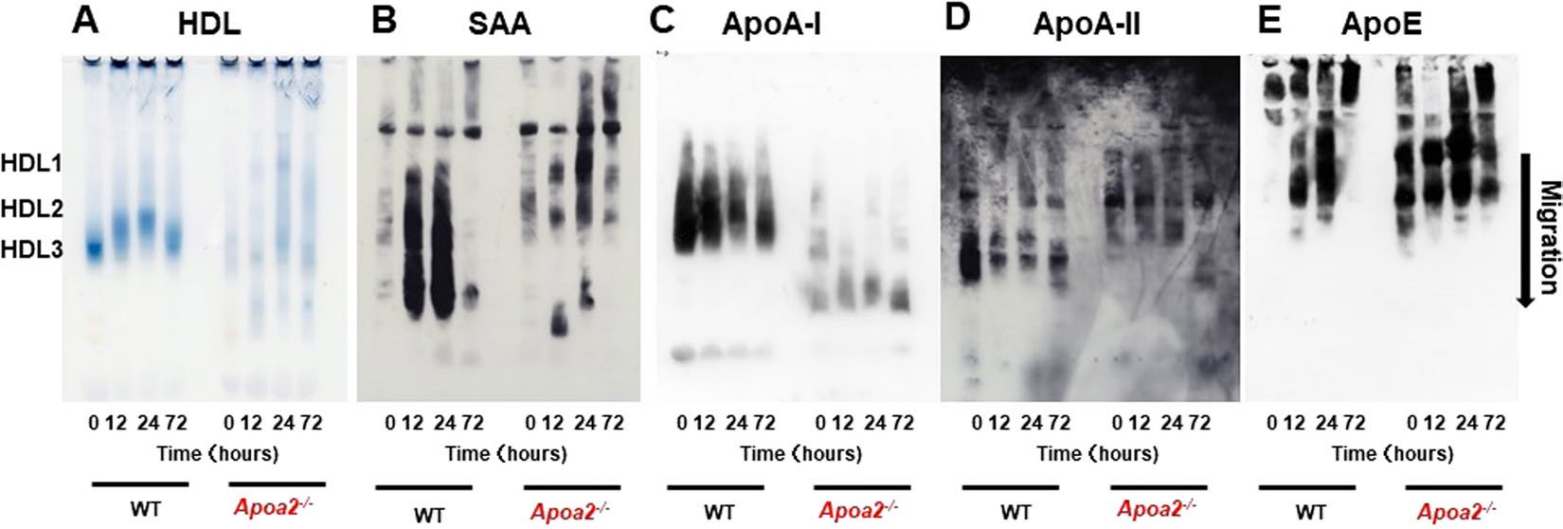
ApoA-II deficiency resulted in the disruption of HDL structure and redistribution of SAA during APR. HDL particle size and distributions of major apolipoproteins among HDL species were analyzed by non-denaturing 5–15% PAGE. To detect HDL particle size, 5 μl of pooled serum obtained from WT or *Apoa2*^*−/−*^ mice during APR at 0 h, 12 h, 1 d and 3 d after co-injection with AgNO_3_ and AA fibrils were pre-stained with Sudan Black B and applied to non-denaturing 5–15% PAGE (**A**). SAA (**B**), ApoA-I (**C**), ApoA-II (**D**), and ApoE (**E**) protein distributions among lipoprotein species were detected by Western blot analysis with specific antisera following non-denaturing 5–15% PAGE of 1 μl pooled serum of mice. All blots were obtained under the same experimental conditions, and cropped images of the blots are shown.

To elucidate the effect of ApoA-II deficiency on lipoprotein particles and the distribution of major apolipoproteins containing SAA, we analyzed HDL particle size by non-denaturing gradient PAGE with serum pre-stained by Sudan Black B (Fig. 7A). Further, we performed Western blot analysis of serum amyloidogenic apolipoproteins (SAA, ApoA-I, ApoA-II and ApoE) (Fig. 7B–E). We marked the size classes of HDL_1_, HDL_2_, and HDL_3_ based on the distributions of ApoA-I in WT mice according to our previously reported results^29,32^. The predominant form of HDL in WT mice was HDL_3_. The HDL particle size increased within 24 h of APR, before returning to the pre-inflammatory size by 72 h. In pre-inflammatory *Apoa2*^*−/−*^ mice, the HDL band was weak, broader and smaller corresponding to HDL_3_ (Fig. 7A), while during APR, the density, size and distribution of HDL particles increased. Without inflammatory stimulation, SAA was not detected by Western blot in WT and *Apoa2*^*−/−*^ mice (Fig. 7B). During APR, SAA increased dramatically and was mainly present within HDL_3_, HDL_2_ and HDL_1_, with the HDLs smaller than HDL_3_ in WT mice. However, SAA was found in HDL_2_ and HDL_1_ in *Apoa2*^*−/−*^ mice, as well as in HDLs smaller than HDL_3_ and larger lipoproteins. ApoA-I was mainly found within HDL_3_ and HDL_2_ for WT mice, and partially in HDL_1_, as determined by PAGE. During APR, the level of ApoA-I decreased gradually and was largely found in HDL_3_ and HDL_2_, and the particle size only increased slightly. In *Apoa2*^*−/−*^ mice, the amount of ApoA-I was dramatically decreased, and was mainly found in HDL particles smaller than HDL_3_ in normal and APR states (Fig. 7C). ApoA-II was distributed mainly in smaller HDL_3_ compared with ApoA-I containing HDL_3_ in WT mice. The amount of ApoA-II decreased and the size of HDL containing ApoA-II increased slightly during APR (Fig. 7D). There was no ApoA-II in *Apoa2*^*−/−*^ mice, as expected. ApoE was distributed widely, and found in lipoproteins ranging from HDL_1_ to larger lipoprotein particles in both WT mice and *Apoa2*^*−/−*^ mice. These observations suggest that ApoA-II deficiency resulted in the disruption of HDL structure and redistribution of elevated SAA and ApoA-I during APR. There was no obvious influence on the distribution of ApoE in this study.

**Figure 8:**
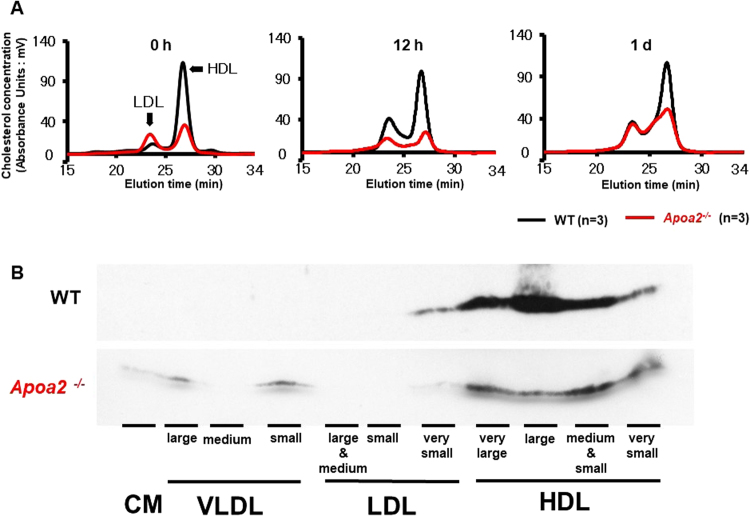
SAA was more widely distributed in various lipoprotein particles in *Apoa2*^*−/−*^ mice as determined by HPLC analysis. Pooled serum (5 µl) from WT and *Apoa2*^*−/−*^ mice at 0 h, 12 h and 1 d after co-injection with AgNO_3_ and AA fibrils was analyzed by HPLC for serum lipoproteins (**A**). Isolated HPLC fractions of ~30 μl pooled serum from WT and *Apoa2*^*−/−*^ mice during APR at 12 h was separated by SDS-PAGE, and SAA protein was determined by Western blot analysis (**B**). All blots were obtained under the same experimental conditions, and cropped images of the blots are shown.

To further confirm these results, pooled sera from WT and *Apoa2*^*−/−*^ mice isolated at 0 h, 12 h, and 1 d after co-injection were analyzed with a HPLC gel filtration system (Fig. 8A). Under normal conditions, HDL cholesterol levels were markedly decreased in *Apoa2*^*−/−*^ mice, while low density lipoprotein (LDL) cholesterol levels were increased compared with WT mice. During APR at 1 d, WT and *Apoa2*^*−/−*^ mice showed an increase in lipoprotein cholesterol levels in LDL and very large HDL. Western blot analysis of isolated HPLC fractions showed that SAA protein was clearly associated with very small to very large HDL in WT mice, as well as very small LDL. However, in *Apoa2*^*−/−*^ mice, SAA distributed more widely, and was found in very small to very large HDL, very small LDL, very low density lipoprotein (VLDL) and chylomicron (CM), compared with WT mice (Fig. 8B).

## Discussion

In this study, we investigated whether apoA-II could influence the metabolism of SAA and HDL and formation of AA amyloidosis in mice. Our previous study showed that serum ApoA-II and SAA interact with AApoAII and AA amyloid fibrils, and facilitate amyloid formation in R1.P1-*Apoa2*^*c*^ mice^28^. SAA protein cross-reacts with pre-existing AApoAII amyloid fibrils and complements the seeding effect of AA fibrils to SAA, increasing AA amyloidosis^28^. On the other hand, increased SAA expression during APR reduces the serum concentration of ApoA-II and suppresses AApoAII amyloidosis. However, there are no studies demonstrating the effects of deficiency and overexpression of ApoA-II on SAA metabolism and AA amyloidosis.

We employed *Apoa2*^*−/−*^ and *Apoa2*Tg mice to explore the mechanism by which ApoA-II influences SAA metabolism and AA amyloidosis. After co-injection of AgNO_3_ (inflammation inducer) and AA fibrils (seed), we showed a significant effect of ApoA-II on elevation of serum SAA levels and AA amyloidosis (Figs 1, 2, 3 and 5). To further elucidate the mechanism of SAA suppression in *Apoa2*^*−/−*^ mice, we analyzed hepatic SAA mRNA expression by real time PCR. Expression of hepatic SAA mRNA in *Apoa2*^*−/−*^ mice was significantly lower than in WT mice in both pre-inflammatory and inflammatory states (Fig. 4). During inflammation, hepatic synthesis of SAA is induced by the macrophage-secreted cytokines IL-6, IL-1, and TNFα via an Nf-κB-dependent pathway. Pathological investigation showed that *Apoa2*^*−/−*^ mice experienced less lung tissue damage and inflammatory cell infiltration during APR. Moreover, the lungs of *Apoa2*^*−/−*^ mice showed quicker recovery from inflammatory damage than WT mice (Fig. 5A to K). However, the impact of elevated SAA on inflammation and tissue damage remains controversial^8^. It has been shown that ApoA-II plays multiple and controversial roles in influencing inflammation^23,34^. Some studies have shown that overexpression of ApoA-II could confer pro-inflammatory properties to HDL by reducing protection against LDL oxidation and stimulating lipid hyperoxidation and monocyte transmigration in mice^34^ and that ApoA-II may maintain host responses to lipopolysaccharide (LPS) by suppressing inhibitory activity of LPS binding protein^35^. In contrast, other studies have suggested an anti-inflammatory effect of ApoA-II in humans^36^. Macrophages and monocytes activated by inflammation may cleave the C-terminal part of SAA and induce AA amyloid fibril formation^37^. Macrophages may also resorb amyloid deposits^37–39^. Thus, inflammation may change the microenvironment of tissues in which amyloid deposits form, and may modulate the progression of AA amyloidosis. Our study demonstrates that ApoA-II provokes inflammation and increases cytokines and macrophages. Further research is required to validate these results.

In mouse AA amyloidosis, amyloid deposition occurs independent of inflammation, while the time and degree of amyloid deposition is determined by plasma SAA concentration in transgenic mice^40,41^. In human AA amyloidosis, plasma SAA concentration is a major factor determining amyloid deposition^37^. In other systemic amyloidosis, circulating concentrations of precursor protein determine amyloid deposition. In *Apoa2*Tg mice, an increase in serum ApoA-II levels of 1.26× was shown to lead to accelerated AApoAII amyloid deposition^32^, and reduced ApoA-II concentration was associated with decelerated amyloid deposition by treatment with calorie-restriction^42^. Thus, we believe that a lower SAA concentration suppresses AA amyloidosis in *Apoa2*^*−/−*^ mice, and increased SAA accelerates amyloid deposition in *Apoa2*Tg mice. The genetic background of *Apoa2*Tg mice is the R1.P1-*Apoa2*^*c*^ strain, and elevation of SAA concentration during APR in this strain was less obvious compared with C57BL/6 J mice that have a genetic background of *Apoa2*^*−/−*^ (Figs 3 and 6). Due to the different genetic background and the relatively smaller number of *Apoa2*Tg mice examined compared with *Apoa2*^*−/−*^ mice, we plan to examine transgenic ApoA-II mice more intensively in future studies to provide additional support for our findings. We believe that a decrease in serum SAA levels in *Apoa2*^*−/−*^ mice was associated with suppression of amyloid deposition and tissue damage. However, there is a possibility that structural changes in AA amyloid fibrils of *Apoa2*^*−/−*^ mice could affect amyloid deposition, and will be the topic of future amyloidosis research.

During APR, plasma proteins increase by at least 25% and the major acute-phase proteins include C-reactive protein (CRP), SAA and fibrinogen^43^. Increased SAA circulates with HDL and results in redistribution of HDL and its apolipoproteins. HDL contains several apolipoproteins, such as SAA, ApoA-I, ApoA-II, ApoA-IV, C-II, C-III and ApoE, which are currently considered to be amyloidogenic or amyloid-associated proteins^3,44^. The most abundant HDL apolipoprotein that is impacted during APR is ApoA-I^45–47^. A number of reports have found marked decreases in serum ApoA-I during APR^48–51^, and acute phase HDL is larger in size than normal HDL_3_, with the radius extending into the HDL_2_ range^52^. In contrast, other studies reported no changes in HDL cholesterol or ApoA-I levels when SAA was increased to levels comparable to those during infection or inflammation^46,53^. In our study, HDL particle size was increased and ApoA-I and ApoA-II decreased during APR (Fig. 7). Some reports have revealed decreased hepatic expression and serum concentrations of ApoA-II during the acute phase^28,43^. Interestingly, our observation showed that the increased SAA was widely distributed during APR, from LDL, HDL_1_, HDL_3_ to very small HDL, while ApoA-I and ApoA-II were not found in LDL and very small HDL (Figs 7 and 8).

Despite these results, the role of ApoA-II in HDL metabolism remains unclear^22^. We sought to elucidate the effect of ApoA-II deficiency on the distribution of lipoprotein particles and other apolipoproteins during APR. In *Apoa2*^*−/−*^ mice, lipoproteins were dramatically decreased and widely distributed, from LDL to very small HDL (Figs 7 and 8). ApoA-I was also dramatically deceased, with its distribution ranging from HDL2 to very small HDL. HDL cholesterol in *Apoa2*^*−/−*^ mice exhibited a smaller size compared with that of WT mice (Fig. 8A). ApoA-II-deficient mice have been reported to have dramatically decreased and smaller HDL^54^, consistent with our observations. In contrast, *Apoa1*^*−/−*^ mice showed slight decreases in ApoA-II levels and a larger HDL size in our previous study^29^. During APR, *Apoa2*^*−/−*^ mice showed an increase in lipoprotein levels, mainly from LDL and very large HDL at 24 h (Fig. 8A). Surprisingly, we observed that SAA in *Apoa2*^*−/−*^ mice was more predominantly located in CM, LDL, HDL and very small HDL, as assessed by PAGE and HPLC gel filtration analysis (Fig. 8B). However, ApoA-I was found mainly in very small HDL during APR in *Apoa2*^*−/−*^ mice. Our previous study showed that ApoA-I plays a key role in maintaining ApoA-II distribution and HDL particle size^29^. On the other hand, it has been reported that ApoA-II is an important factor regulating HDL size and the ratio of ApoA-I to ApoA-II in plasma^55^. ApoA-II is more hydrophobic and modulates more strongly the conformation of HDL than ApoA-I^56–58^. Our results revealed that, compared with ApoA-I, ApoA-II is as a stronger regulator of lipoprotein metabolism and apolipoprotein stabilization. Conformational changes of lipoprotein particles may alter the binding strength of SAA to lipoprotein. The extracellular matrix bound by SAA includes heparan sulfate proteoglycan and anti-inflammatory effects of lipoproteins. It has been suggested that conformational changes of lipoproteins may increase the susceptibility to AA amyloidosis^59^.

Our study showed that A-II plays a critical role in the pathogenesis of AA amyloidosis in mice. Important factors associated with the pathogenesis of AA amyloidosis include: (1) serum concentration of SAA, (2) SAA associated circulating lipoprotein structure, and (3) the microenvironment in which SAA forms fibrils and deposits. Recent studies showed that ApoA-II plays multiple roles in regulating the metabolism of plasma HDL and its apolipoproteins, and in prevention or acceleration of cardiovascular disease^23,54,60^. This study sheds light on the relationship between ApoA-II and SAA, and provides new information regarding the pathogenesis of amyloidosis associated with HDL-related proteins. ApoA-II may serve as a therapeutic target for AA. Additional studies are warranted to further explore this important issue.

## Materials and Methods

### Mice and induction of AA amyloidosis

C57BL/6 JJmsSlc mice were obtained from Japan SLC Inc. (Hamamatsu, Japan). C57BL/6-B6.129P2-*Apoa1*^*tm1Unc*^*/J* and C57BL/6-B6.129S4-*Apoa2*^*tm1Bres*^*/J* mice were purchased from Jackson Laboratories (Bar Harbor, ME). The ApoA-I deficient (*Apoa1*^*−/−*^) and ApoA-II deficient (*Apoa2*^*−/−*^) strains were produced by backcrossing the *Apoa1*^*tm1Unc*^ and *Apoa2*^*tm1Bres*^ allele 10 times to C57BL/6J mice. *Apoa2*^*c*^ transgenic mice (*Apoa2*Tg) were generated on a genetic background of congenic SAMR1.SAMP1- *Apoa2*^*c*^ (R1.P1- *Apoa2*^*c*^) mice using backcross procedures^32^. Mice were maintained under specific pathogen free (SPF) conditions at 24 ± 2 °C with a light-controlled regimen (12 hours light/dark cycle) in the Division of Laboratory Animal Research, Research Center for Supports to Advanced Science, Shinshu University. A commercial diet (MF; Oriental Yeast, Tokyo, Japan) and tap water were provided *ad libitum*. All experiments using animals were performed with the approval of the Committee for Animal Experiments of Shinshu University under permit numbers 270016 (from 2015) and 280014 (from 2016), and with the approval of the Shinshu University Safety Committee for Recombinant DNA Experiments under permit numbers 15-007 (from 2015) and 16–016 (from 2016). Approved protocols were strictly followed.

Two-month-old male mice were subjected to the experiments for APR and AA amyloidosis. Mice were co-injected with AgNO_3_ (1%, 0.5 ml, subcutaneous injection) and AA amyloid fibrils (100 μg, intravenous injection). Blood samples were collected at 6 and 12 hours (h), and at 1, 2, 3, 5, 7 and 10 days (d) after injection. Mice were sacrificed by cardiac puncture under isoflurane anesthesia 12 h, 1 d, 3 d, and 10 d after co-injection, and major organs were fixed in 10% neutral buffered formalin and embedded in paraffin.

### Isolation of amyloid fibrils

AA fibrils were isolated from the pooled livers and spleens of C57BL/6 J mice with severe AA amyloidosis. Amyloid fibril fractions were isolated by Pras’ method with some modification^32^. Isolated amyloid fibrils were suspended in deionized/distilled water (DW) at a concentration of 1.0 mg/ml and were stored at −70 °C until use. We placed 1 ml of this mixture into a 1.5 ml Eppendorf tube and sonicated on ice for 30 seconds (s) with an ultrasonic homogenizer VP-5S (Tietech Co., Ltd., Tokyo, Japan) at maximum power. This procedure was repeated 5 times at 30 s intervals. Sonicated AA fibril samples were used immediately.

### Detection of amyloid deposition and inflammation in mice

Mouse organs embedded in paraffin were sliced into 4 μm sections and stained with Mayer’s hematoxylin/eosin (HE) or Congo red. Amyloid deposition was identified under polarizing microscopy by green birefringence in tissue sections stained by Congo red^61^. AA fibrils were also identified by IHC analysis using the avidin–biotin horseradish peroxidase complex method with specific rabbit antiserum against mouse AA, which was produced against guanidine hydrochloride-denatured AA fibrils in our laboratory^62^. The antiserum reacts specifically with AA amyloid deposits in IHC analysis and serum SAA protein in Western blot analysis^32,63,64^. For quantitative IHC analysis, 3 areas in each liver and spleen section were randomly captured and the ratios of positively stained areas with anti-AA antiserum to the whole section areas were calculated using an image processing program (NIH Image J software, version 1.61). The intensity of the AA deposit was also determined semi-quantitatively using the amyloid score (AS) and amyloid index (AI). As described previously, the AI is the average of the AS graded from 0 to 4 in the 7 organs examined (liver, spleen, skin, heart, stomach, small intestine and tongue) in sections stained with Congo red and observed under polarizing microscopy^63^. Two blinded observers, who had no information regarding the tissues, graded the AS.

Two blinded observers, who had no information regarding the tissue, observed pathological damage and inflammatory cell infiltration in tissue specimens stained with HE. We evaluated the damage score by quantifying the area of inflammatory cells and pulmonary edema. The inflammatory cell infiltration area was scored as follows: 0 (<5%), 1 (5–25%), 2 (25–50%), 3 (>50%). The area of pulmonary edema was scored as: 0 (absent), 1 (<25%), 2 (25–50%), 3 (>50%). The sums of these two area scores represented final damage scores for statistical analyses.

### Serum concentration of SAA

We isolated serum by centrifugation of blood at 3,000 g for 20 min at 4 °C. The serum (0.5 or 1 μl) was boiled in sample buffer (2% SDS, 12% glycerol, 62.5 mM Tris pH 6.8, 10% 2-mercaptoethanol, 0.02% bromphenol blue) and separated by electrophoresis at 2 mA for 2 h, followed by 20 mA for 4 h on Tris-Tricine/SDS-16.5% polyacrylamide gel electrophoresis (SDS-PAGE). Proteins in the gels were transferred electrophoretically to polyvinylidine difluoride (PVDF) membranes. Proteins on the membranes were reacted with anti-AA antiserum (1:3000), followed by peroxidase-conjugated anti-rabbit IgG solution (1:3000) (Cell Signaling Technology, Massachusetts, USA)^63^. Immunoreactive proteins were visualized with the enhanced chemiluminescence (ECL) system (Amersham Biosciences, Buckinghamshire, England) with X-ray film (Amersham Biosciences). For quantification, Western blot images were captured and analyzed using Scion Image version 4.0.3.2. Serum SAA levels were represented as ratios to pooled standard serum (ratio = 1.0) isolated from C57BL/6 J mice at 1 d after co-injection of AgNO_3_ and AA amyloid fibrils.

### *Saa1* and *Saa2* mRNA expression in the liver

Total RNA was extracted from the livers of C57BL/6 J and *Apoa2*^*−/−*^ mice at 0 h, 12 h and 1 d after co-injection of AgNO_3_ and AA amyloid fibrils, using TRIZOL Reagent (Invitrogen, Carlsbad CA), followed by treatment with DNA-Free reagent (Ambion, Austin TX) to remove contaminating DNA. Total RNA was then subjected to reverse transcription using an Omniscript RT kit with random primers (Applied Biosystems, Foster CA). Quantitative real-time PCR analysis was carried out using an ABI PRISM 7500 Sequence Detection System (Applied Biosystems Foster CA) with SYBR Green (Takara Bio, Tokyo, Japan), and values were normalized with respect to β-actin. We designed primers which detect both *Saa1* and *Saa2* mRNA. The following primers were used: *Saa1/2-F: 5*′*-AGTGGCAAAGACCCCAATTA-*3′, *Saa1/2-R: 5*′*-GGCAGTCCAGGAGGTCTGTA-*3′; β-actin-F: 5′-GACAGGATGCAGAAGGAGATTACT-*3*′ and β-actin-R: 5′-TGATCCACATCTGCTGGAAGGT-*3*′.

### Serum lipoprotein quantity, particle size, and distribution of apolipoproteins

To determine HDL particle size, serum (5 μl for C57BL/6 J mice and *Apoa2*^*−/−*^ mice) samples were pre-stained for lipids with Sudan Black B and electrophoresed on a non-denaturing PAGE gel with a 5 to 15% linear polyacrylamide gradient. Electrophoresis was carried out at 25 mA for 2 h^32,33^. The distribution of ApoA-I, ApoA-II, ApoE, and SAA protein among the various lipoprotein particles was determined by Western blot analysis of 1 μl serum separated by non-denaturing PAGE. Antibodies used included: anti-mouse AA (1:3000)^62^, rabbit anti-mouse ApoA-I (1:3000)^65,66^, rabbit anti-mouse ApoA-II (1:200) (sc-366255, Santa Cruz Biotechnology, Dallas, TX) and goat anti-apoE antiserum (1:200) (sc-6384, Santa Cruz Biotechnology). Subsequently, membranes were incubated with anti-rabbit IgG solution (1:3000) or donkey anti-goat IgG-peroxidase conjugated solution (1:3000) (sc-2020, Santa Cruz Biotechnology). To further determine the cholesterol profiles in serum lipoproteins, pooled sera from five C57BL/6 J mice and six *Apoa2*^*−/−*^ mice treated with AgNO_3_ and AA fibrils were analyzed by dual-detection high-performance liquid chromatography (HPLC) (Liposearch System, Skylight Biotech, Inc., Akita, Japan)^67^. In addition, isolated HPLC fractions with different particle diameters (ranging from 7.6 nm to >80 nm) from 30 µl pooled serum were concentrated. SAA protein in each fraction was detected by Western blot analysis after separation by 16.5% SDS-PAGE.

### Statistical analysis

The SPSS 16.0 software package (Abacus Concepts, Berkley, CA) was used to analyze the data. All data are presented as mean ± SEM. Significant differences in real time PCR and AA IHC positive areas were examined by unpaired Student’s t-test or the Tukey–Kramer method for multiple testing. Because the AI and damage score are nonlinear indexes, significant differences were examined using the nonparametric Mann-Whitney U-test. A two-tailed P value of <0.05 was considered to be statistically significant.

## Electronic supplementary material

Dataset1
