# Supplementary material for: Apolipoprotein A-II induces acute-phase response associated AA amyloidosis in mice through conformational changes of plasma lipoprotein structure
Source: Sci Rep. 2018 Apr 4;8:5620. doi: 10.1038/s41598-018-23755-y (PMC5884826; doi:10.1038/s41598-018-23755-y)
Supplement: Supplementary file 1 — Dataset1 [file 41598_2018_23755_MOESM1_ESM.docx]

**Apolipoprotein A-II induces acute-phase response associated AA amyloidosis in mice through conformational changes** **of plasma lipoprotein structure**

**Mu YANG^1,2^, Yingye LIU^1,3^, Jian DAI^1^, Lin LI^1^, Xin DING^1^, Zhe XU^1^, Masayuki MORI^1,4^, Hiroki MIYAHARA^1^, Jinko SAWASHITA^1,5^ and Keiichi HIGUCHI^1,5^**

**Supplementary Figure**

**
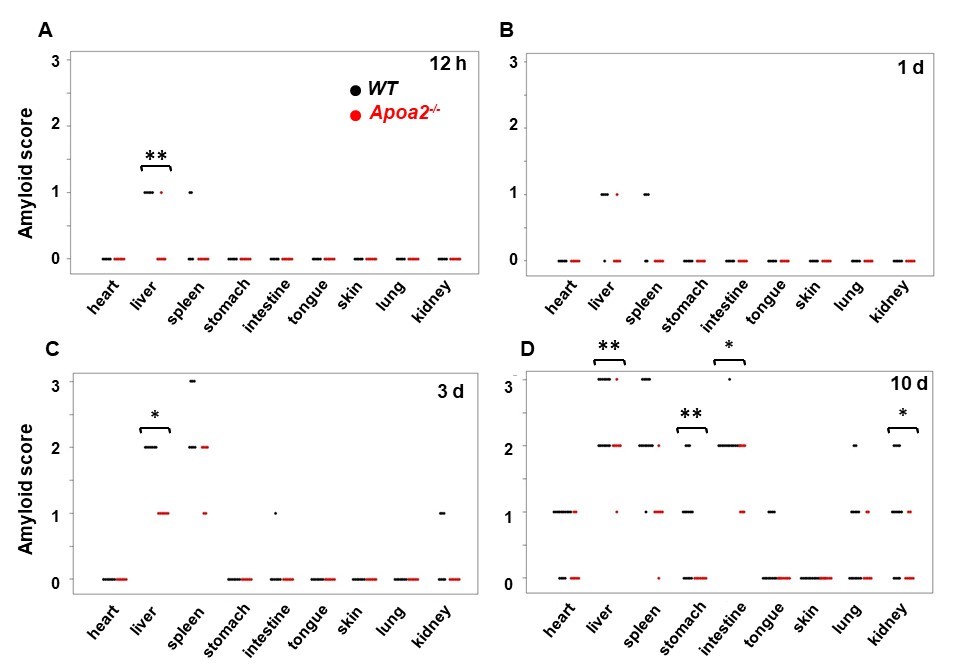
**

**Figure S1**

The degree of AA amyloid deposition in mouse organs (heart, liver, spleen, stomach, intestine, tongue, skin, lung and kidney). The degree of amyloid deposition in each organ (amyloid score, AS) was determined in WT and *Apoa2^-/-^* mice at (A) 12 h, (B) 1 d, (C) 3 d and (D) 10 d after co-injection of AgNO_3_ and AA fibrils. The AS of each mouse is presented. ASs of the liver in *Apoa2^-/-^* mice were suppressed at 12 h and 3 d. After 10 d, The AS in *Apoa2^-/-^* mice was lower than in WT mice in the spleen, stomach, intestine and kidney. *, ** significantly different between WT and *Apoa2^-/-^* mice, P<0.05, P<0.01 (Mann-Whitney U-test).
